# A Systematic Analysis on the Genes and Their Interaction Underlying the Comorbidity of Alzheimer's Disease and Major Depressive Disorder

**DOI:** 10.3389/fnagi.2021.789698

**Published:** 2022-01-20

**Authors:** Pan Guo, Shasha Chen, Hao Wang, Yaogang Wang, Ju Wang

**Affiliations:** ^1^School of Biomedical Engineering, Tianjin Medical University, Tianjin, China; ^2^School of Public Health, Tianjin Medical University, Tianjin, China

**Keywords:** Alzheimer's disease, major depressive disorder, comorbidity, pathway cross-talk, functional enrichment analysis

## Abstract

**Background:**

During the past years, clinical and epidemiological studies have indicated a close relationship between Alzheimer's disease (AD) and other mental disorders like major depressive disorder (MDD). At the same time, a number of genes genetically associated with AD or MDD have been detected. However, our knowledge on the mechanisms that link the two disorders is still incomplete, and controversies exist. In such a situation, a systematic analysis on these genes could provide clues to understand the molecular features of two disorders and their comorbidity.

**Methods:**

In this study, we compiled the genes reported to be associated with AD or MDD by a comprehensive search of human genetic studies and genes curated in disease-related database. Then, we investigated the features of the shared genes between AD and MDD using the functional enrichment analysis. Furthermore, the major biochemical pathways enriched in the AD- or MDD-associated genes were identified, and the cross talks between the pathways were analyzed. In addition, novel candidate genes related to AD and MDD were predicted in the context of human protein-protein interactome.

**Results:**

We obtained 650 AD-associated genes, 447 MDD-associated genes, and 77 shared genes between AD and MDD. The functional analysis revealed that biological processes involved in cognition, neural development, synaptic transmission, and immune-related processes were enriched in the common genes, indicating a complex mechanism underlying the comorbidity of the two diseases. In addition, we conducted the pathway enrichment analysis and found 102 shared pathways between AD and MDD, which involved in neuronal development, endocrine, cell growth, and immune response. By using the pathway cross-talk analysis, we found that these pathways could be roughly clustered into four modules, i.e., the immune response-related module, the neurodevelopmental module, the cancer or cell growth module, and the endocrine module. Furthermore, we obtained 37 novel candidate genes potentially related to AD and MDD with node degrees > 5.0 by mapping the shared genes to human protein-protein interaction network (PPIN). Finally, we found that 37 novel candidate genes are significantly expressed in the brain.

**Conclusion:**

These results indicated shared biological processes and pathways between AD and MDD and provided hints for the comorbidity of AD and MDD.

## Introduction

As the most common type of dementia, Alzheimer's disease (AD) affects ~10% of people of 65 years or older (Blennow et al., 2006). Although cognitive impairment is the core feature of this disease, neuropsychiatric symptoms appear commonly in patients with AD and are persistent (Fernández et al., 2010; Kales et al., 2015). Depression and depression symptoms are among the most common symptoms in patients with AD and may be the early symptoms of the dementing process (Lyketsos et al., 1997b). Depression or depressive symptoms may affect as much as 50% of patients with AD (Starkstein et al., 2005), and about 20–30% of patients with AD are influenced by major depressive disorder (MDD) (Ballard et al., 1996; Enache et al., 2011). The continuous increase of aging populations across the world has led to an increasing prevalence of cognitive impairment, which has become a major problem both clinically and socially (Prince et al., 2016; The Alzheimer's Association, 2020). Specifically, AD and MDD are both common diseases of the elderly and are often comorbidity (Lee and Lyketsos, 2003). Thus, exploring the association between AD and MDD will not only help us to elucidate the neurobiological mechanisms underlying the two diseases but also be useful for developing better therapeutic approaches for them.

However, the relationship between AD and MDD is complex and not well-understood. Depression and depressive symptoms not only are common and highly persistent in patients with AD but also may increase the risk of functional decline and behavioral disturbance (Migliorelli et al., 1995; Ballard et al., 1996; Lyketsos et al., 1997b; Olin et al., 2002). It is reported that depressed patients may be more likely to develop AD than people without the symptoms (Saczynski et al., 2010). In contrast, no connection between the two conditions has been found in some studies. For example, in a cohort including 4,615 elderly Canadians, the history of depression is not significantly associated with the risk of dementia (Lindsay et al., 2002); in another study based on people aged 85 years or over, it is reported that depression is associated with cognitive impairment, but depression alone may not increase the risk of cognitive decline (Vinkers et al., 2004).

At the molecular level, both AD and MDD are diseases influenced by multiple genetic factors. During the past years, a number of genes, such as apolipoprotein E (APOE), ATP binding cassette subfamily A member 7 (ABCA7), and others (Steinberg et al., 2015; Serrano-Pozo et al., 2021), have been found to be genetically associated with AD. Some studies found the shared genetic risk factors could partially explain the observed association between AD and MDD. A positive association between ApoE4 allele and late-onset depression has been reported in some studies (Krishnan et al., 1996; Kim et al., 2002). However, in other studies, no association between ApoE4 allele and depression in patients with AD was detected (Lyketsos et al., 1997a; Scarmeas et al., 2002). Since both AD and MDD are cognitive disorders, hippocampus plays a principal role in memory deficits in patients with AD and MDD. In patients with AD, the decrease in hippocampal volume is related to cognitive decline and other symptoms of AD neuropathology (Jack et al., 1999; van der Flier and Scheltens, 2009). Furthermore, a correlation between hippocampal volume loss in the presence of ApoE allele epsilon4 and decreased cerebrospinal fluid beta-amyloid, the biomarkers of AD, has been observed (Schuff et al., 2009). Structural studies also have found that hippocampal volume is smaller in patients with MDD compared with people without depression, and hippocampal atrophy is suggested as a biomarker for cognitive decline in patients with MDD (Taylor et al., 2014; Schmaal et al., 2017). Amyloid-β (Aβ) deposition is an important pathological change in the AD brain, and it has been found that lifelong major depression is associated with the deposition of Aβ in the brain (Wu et al., 2014). Abnormal neuroinflammation is related to the pathological features of AD and has a negative impact on cognitive function (Cai et al., 2019; Passamonti et al., 2019). Studies have revealed an association between neuroinflammation and the pathogenesis of MDD or AD (Barber, 2011); however, the impact of neuroinflammation is much larger and more complicated, and further exploration is necessary. The study found that late-life major depression with dementia was significantly associated with a neuropathologic diagnosis of AD (Sweet et al., 2004). However, some studies report no association between lower hippocampal volume and AD pathology in late-life depression (De Winter et al., 2017).

Therefore, the correlation between AD and depression is still not fully understood. Obviously, it is necessary to further explore the biological mechanisms underlying the comorbidity of AD and MDD, which may help us design better therapeutic approach for the diseases.

In current study, we comprehensively collected genes related to AD or MDD. We next employed the functional analysis to detect the major biological processes and biochemical pathways and further investigated the interactions among the significantly enriched pathways. Of significance, we predicted some novel candidate genes potentially related to AD and MDD by analyzing human protein-protein interaction network (PPIN). This study will provide valuable clues for understanding the molecular mechanisms underlying the comorbidity of AD and MDD.

## Materials and Methods

### Data Collection

The DisGeNET (https://www.disgenet.org/) is a database that comprehensively integrates human disease-associated genes and their variants (Piñero et al., 2020). It compiles data from expert curated repositories, text mining data extracted from scientific literature, experimentally validated data, and referred data. A gene with a gene-disease associations (GDA) score above 0.2 indicates a strong association with the disease. In the current study, only genes with a GDA score >0.2 were included in the analysis. A list of 146 AD-associated genes was retrieved from DisGeNET. Furthermore, in an earlier study (Hu et al., 2017), we collected 430 genes related to AD from reports deposited at PubMed (https://pubmed.ncbi.nlm.nih.gov/). These genes were identified from either candidate gene association studies (CGAS) or GWAS on human samples. This gene set was updated to include the genes reported in the recent publications, and a total of 587 genes associated with AD were obtained. Then, this gene list was merged with those retrieved from DisGeNET, a list of 650 unique AD-associated genes were left.

For MDD, we retrieved 266 genes with a GDA score >0.2 from the DisGeNET database. Additionally, Fan et al. (2020) compiled a list of 255 MDD-associated genes. By integrating the two gene sets, we obtained a total of 447 unique MDD-associated genes.

### Functional Enrichment Analysis

The gene ontology (GO) and pathway enriched in the shared genes were annotated using the WebGestalt (http://www.webgestalt.org/option.php) (Zhang et al., 2005; Wang et al., 2013). The Benjamini and Hochberg (B&H) method for false discovery rate correction was applied to correct the original *p*-value. WebGestalt is an online-based system to integrate and visualize gene set in various biological contexts including GO. Only the GO terms with FDR value smaller than 0.05 were chosen as significantly enriched terms in our study. The biochemical pathways enriched in the 650 AD-associated genes and the 447 MDD-associated genes were identified by ToppGene (https://toppgene.cchmc.org/enrichment.jsp) (Chen et al., 2009), which is an online tool that integrates different pathway databases such as Kyoto Encyclopedia of Genes and Genomes (KEGG) (Aoki-Kinoshita and Kanehisa, 2007), Pathway Interaction Database (PID) (Schaefer et al., 2009), and Reactome (Croft et al., 2011). For the pathway analysis, only KEGG was chosen as the pathway database, and pathway with FDR < 0.05 was defined as significant pathway.

### Cross-Talk Analysis Among Pathways

Here, we first obtained pathways enriched in the AD-associated genes or MDD-associated genes, respectively. We selected the overlapping pathways of AD and MDD to construct the pathway cross-talk network by integrating AD-associated genes and MDD-associated genes. As described in our previous report (Guo et al., 2021), we adopted two measurements (Jia et al., 2011; Liu et al., 2015), i.e., the Jaccard Coefficient (JC) $=|\frac{A⋂B}{A\cup B}|$ and the Overlap Coefficient (OC) $=\frac{\left|A⋂B\right|}{\min\left(\left|A|,|B\right|\right)}$, with A and B being the lists of genes included in the two tested pathways, and |A| and |B| representing the number of genes contained in the two pathways. Finally, the cross talk between pathways was constructed and visualized using the Cytoscape 3.7.1 software (Kohl et al., 2011).

### Prediction of New Candidate Genes Related to AD and MDD

In this study, the PPIN data were obtained from the Protein Interaction Network Analysis (PINA) database (Cowley et al., 2012) by pooling and curating the unique physical interaction information from six main public protein interaction databases, i.e., BioGRID, IntAct, DIP, MINT, MIPS/MPact, and HPRD. In the meantime, Menche et al. (2015) reported an interactome for human that contained 141,296 edges among 13,460 nodes. Later, we merged the two interactomes data by excluding the self-interaction and redundant pairs. We excluded ubiquitin-C, B, and D (i.e., UBC, UBB, and UBD) from this network because of the non-specific binding of ubiquitin to proteins for degradation (Ferrari et al., 2018). Finally, we obtained a relatively complete human physical interactome, which included 15,435 genes/protein and 218,161 interactions (Supplementary Material 1).

We mapped the 650 AD-associated genes to the PPIN and obtained an AD-specific network containing 9,089 genes/protein and 32,065 interactions. Similarly, by mapping the 447 MDD-associated genes to the PPIN, we obtained an MDD-specific network containing 6,541 genes/protein and 18,221 interactions. Then, the irrelevant interactions were excluded by merging the AD-specific network and MDD-specific network into a single disease network, which contained 10,203 genes/protein and 45,385 interactions. Finally, the AD-specific network was compared against the MDD-specific network, and the overlapping part was extracted, and the node degree of each node in this overlapping network was calculated with the disease network as background *via* the “Network Analyzer” (Assenov et al., 2008) plug-in in the Cytoscape software. After removing AD-associated genes and MDD-associated genes, the remaining genes could be expected to be novel candidate genes for both diseases.

### Analysis of Temporal and Cell Type-Specific Expression

The brain is composed of different but interdependent regions, with which being made up of many different types of cells performing specific functions. The precise spatial and temporal regulation of gene expression in these cells plays key roles in the function of the brain. To explore the biological features of new candidate genes in a specific brain cell type, brain region, and developmental stages, the corresponding gene expression pattern was analyzed. Cell-type specific expression analysis (CSEA; http://genetics.wustl.edu/jdlab/csea-tool-2/) (Xu et al., 2014) was used to compare the expression pattern of new candidate genes in different brain cells or brain regions. The Fisher's exact test was performed across the cell types and developmental stages at a specificity index threshold (pSI) of 0.05. This threshold measures the possibility of the expression of a specific gene in the given cell type, brain region, and developmental stage.

## Results

### Susceptibility Genes of AD and MDD

By searching the available genetic studies on AD and collecting the genes curated in disease-related database DisGeNET, we compiled a list of 650 AD-associated genes (Supplementary Table S1; the gene list is referred to as ADgset, hereafter). Among them were those encoding apolipoprotein, ATP-binding cassette transporters, nicotinic acetylcholine receptors, adrenoceptors, serotonin receptors, dopamine degradation, dopamine receptors, complement receptors, etc. Similarly, we also compiled a list of 447 MDD-associated genes (Supplementary Table S2; the gene list is referred to as MDDgset, hereafter). The MDDgset included genes related to immune regulation, dopamine neurotransmission, serotonin neurotransmission, embryonic development, cellular stress response, etc.

There were 77 shared genes between the two diseases (Table 1), among which included five oxidative stress-related genes (i.e., *HIF1A, HSPA4, MAOA, MAOB, SOD2*), four interleukin genes (i.e., *IL10, IL1B, IL33, IL6*), four neural factors genes (i.e., *BDNF, NGF, NPY, NTF3*), two 5-hydroxytryptamine receptor genes (i.e., *HTR2A, HTR6*), two solute carrier genes (i.e., *SLC6A3, SLC6A4*), two estrogen receptor genes (i.e., *ESR1, ESR2*), two cancer-related genes (i.e., *TNF, TP53*), two LDL receptor-related protein coding genes (i.e., *LRP1, LRP8*), one transcription factors gene (i.e., *TFCP2*), one apolipoprotein gene (i.e., *APOE*), one dopamine receptor gene (i.e., *DRD4*), and others.

**Table 1:** Susceptibility gene shared by Alzheimer's disease (AD) and major depressive disorder (MDD).

**Gene ID**	**Gene symbol**	**Gene name**	**Gene source**
5243	ABCB1	ATP binding cassette subfamily B member 1	Literature/DisGeNet
1636	ACE	Angiotensin I converting enzyme	Literature/DisGeNet
155	ADRB3	Adrenoceptor beta 3	Literature/DisGeNet
348	APOE	Apolipoprotein E	Literature/DisGeNet
406	ARNTL	Aryl hydrocarbon receptor nuclear translocator like	Literature
540	ATP7B	ATPase copper transporting beta	Literature/DisGeNet
613	BCR	BCR, RhoGEF and GTPase activating protein	Literature/DisGeNet
627	BDNF	Brain derived neurotrophic factor	Literature/DisGeNet
6347	CCL2	C-C motif chemokine ligand 2	Literature/DisGeNet
1029	CDKN2A	Cyclin dependent kinase inhibitor 2A	Literature/DisGeNet
89832	CHRFAM7A	CHRNA7 (exons 5–10) and FAM7A (exons A-E) fusion	Literature
9575	CLOCK	Clock circadian regulator	Literature/DisGeNet
26047	CNTNAP2	Contactin associated protein 2	Literature/DisGeNet
1312	COMT	catechol-O-methyltransferase	Literature/DisGeNet
1392	CRH	Corticotropin releasing hormone	DisGeNet
1565	CYP2D6	Cytochrome P450 family 2 subfamily D member 6	Literature/DisGeNet
267012	DAOA	D-amino acid oxidase activator	Literature/DisGeNet
1621	DBH	Dopamine beta-hydroxylase	Literature/DisGeNet
27185	DISC1	Disrupted in schizophrenia 1	Literature/DisGeNet
1740	DLG2	Disks large MAGUK scaffold protein 2	Literature/DisGeNet
1742	DLG4	Disks large MAGUK scaffold protein 4	Literature/DisGeNet
1815	DRD4	Dopamine receptor D4	Literature/DisGeNet
5610	EIF2AK2	Eukaryotic translation initiation factor 2 alpha kinase 2	Literature
2099	ESR1	Estrogen receptor 1	Literature/DisGeNet
2100	ESR2	Estrogen receptor 2	Literature/DisGeNet
79068	FTO	Alpha-ketoglutarate dependent dioxygenase	Literature
2692	GHRHR	Growth hormone releasing hormone receptor	Literature
2784	GNB3	G protein subunit beta 3	Literature/DisGeNet
2904	GRIN2B	Glutamate ionotropic receptor NMDA type subunit 2B	Literature
2932	GSK3B	Glycogen synthase kinase 3 beta	Literature/DisGeNet
3091	HIF1A	Hypoxia inducible factor 1 subunit alpha	Literature/DisGeNet
3240	HP	Haptoglobin	Literature/DisGeNet
3308	HSPA4	Heat shock protein family A (Hsp70) member 4	Literature/DisGeNet
3356	HTR2A	5-hydroxytryptamine receptor 2A	Literature/DisGeNet
3362	HTR6	5-hydroxytryptamine receptor 6	Literature
3586	IL10	Interleukin 10	Literature/DisGeNet
3553	IL1B	Interleukin 1 beta	Literature/DisGeNet
90865	IL33	Interleukin 33	Literature/DisGeNet
3569	IL6	Interleukin 6	Literature/DisGeNet
3952	LEP	Leptin	DisGeNet
4035	LRP1	LDL receptor related protein 1	Literature/DisGeNet
7804	LRP8	LDL receptor related protein 8	Literature/DisGeNet
4128	MAOA	Monoamine oxidase A	Literature/DisGeNet
4129	MAOB	Monoamine oxidase B	DisGeNet
4133	MAP2	Microtubule associated protein 2	DisGeNet
9175	MAP3K13	Mitogen-activated protein kinase 13	Literature
4353	MPO	Myeloperoxidase	Literature/DisGeNet
4524	MTHFR	Methylenetetrahydrofolate reductase	Literature/DisGeNet
4599	MX1	MX dynamin like GTPase 1	Literature
4803	NGF	Nerve growth factor	Literature/DisGeNet
4852	NPY	Neuropeptide Y	Literature/DisGeNet
4908	NTF3	Neurotrophin 3	Literature/DisGeNet
4915	NTRK2	Neurotrophic receptor tyrosine kinase 2	Literature/DisGeNet
84547	PGBD1	piggyBac transposable element derived 1	Literature
65018	PINK1	PTEN induced kinase 1	Literature/DisGeNet
5337	PLD1	Phospholipase D1	Literature
5444	PON1	Paraoxonase 1	Literature
5743	PTGS2	Prostaglandin-endoperoxide synthase 2	Literature/DisGeNet
5649	RELN	Reelin	Literature/DisGeNet
6285	S100B	S100 calcium binding protein B	Literature/DisGeNet
9037	SEMA5A	Semaphorin 5A	Literature
10280	SIGMAR1	Sigma non-opioid intracellular receptor 1	Literature
23411	SIRT1	Sirtuin 1	Literature
6531	SLC6A3	Solute carrier family 6 member 3	Literature
6532	SLC6A4	solute carrier family 6 member 4	Literature/DisGeNet
6648	SOD2	Superoxide dismutase 2	Literature/DisGeNet
6671	SP4	Sp4 transcription factor	Literature
6688	SPI1	Spi-1 proto-oncogene	Literature
6750	SST	Somatostatin	Literature/DisGeNet
7024	TFCP2	Transcription factor CP2	Literature/DisGeNet
7040	TGFB1	Transforming growth factor beta 1	Literature
7124	TNF	Tumor necrosis factor	Literature/DisGeNet
10452	TOMM40	Translocase of outer mitochondrial membrane 40	Literature/DisGeNet
7157	TP53	Tumor protein p53	Literature
7422	VEGFA	Vascular endothelial growth factor A	Literature/DisGeNet
7434	VIPR2	Vasoactive intestinal peptide receptor 2	Literature
91752	ZNF804A	Zinc finger protein 804A	Literature

### Biological Process Enrichment Analysis of Shared Genes

**Figure 1:**
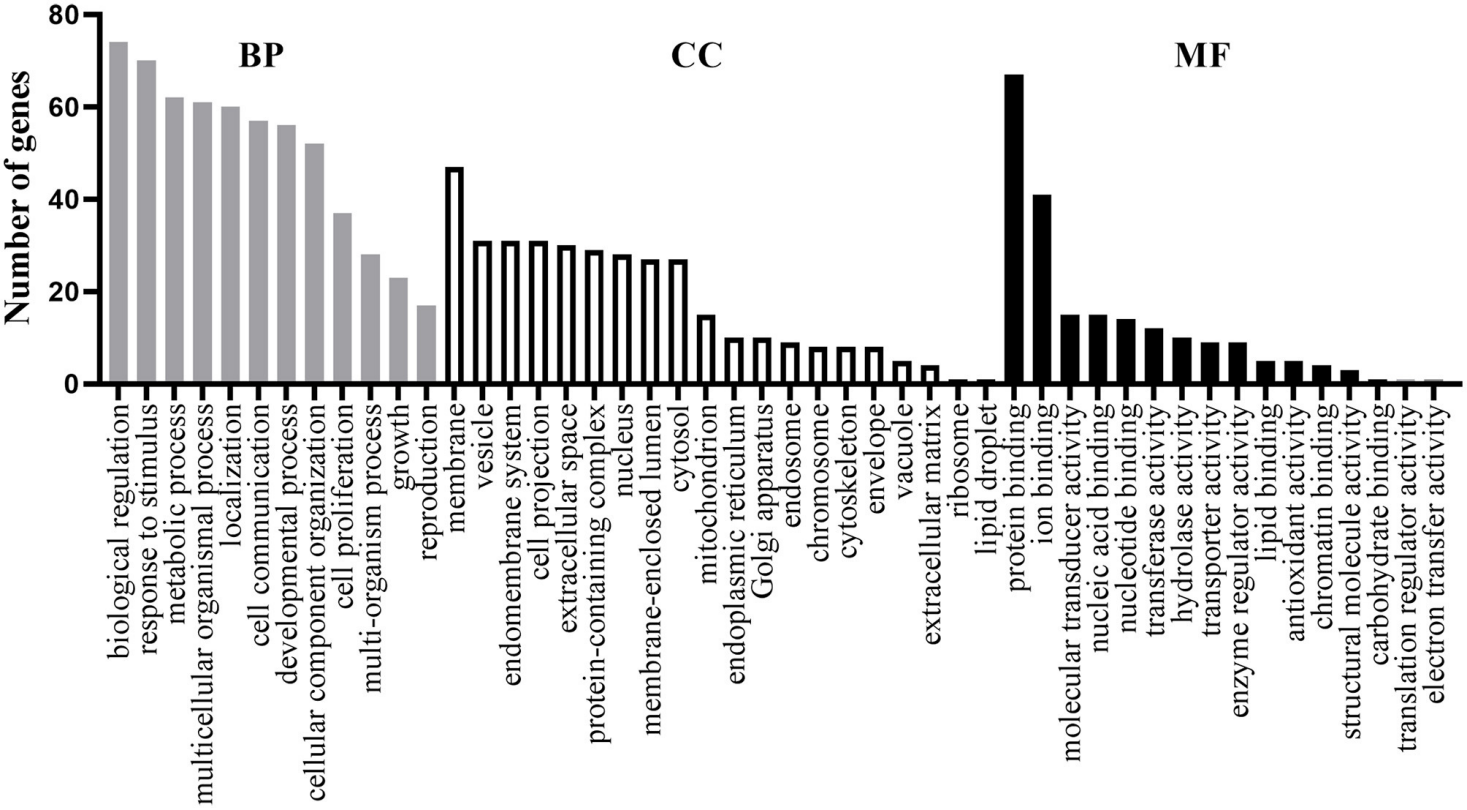
Functional enrichment analysis of the 77 shared genes. Gene ontology (GO) items belonging to biological process (BP), cellular component (CC), and molecular function (MF) are shown in gray, white, and black columns, respectively.

For these 77 shared genes, 74 were significantly enriched in the biological regulation, 47 in membrane, and 67 in the protein binding (Figure 1). Totally, 311 BP GO terms (Supplementary Table S3) were significantly enriched in the shared genes. The most significantly term is cognition, which is in line with the fact that patients with AD and MDD have cognitive impairments (Sierksma et al., 2010). Furthermore, biological processes related to neural development and synaptic transmission were enriched, such as neuron death, dopaminergic neuron differentiation, neuron migration, synaptic transmission, dopaminergic, chemical synaptic transmission, postsynaptic and synaptic transmission, and glutamatergic. Terms associated with drug reactions and metabolic processes were overrepresented, such as response to toxic substance, response to antibiotic, response to oxidative stress, reactive oxygen species metabolic process, hormone metabolic process, receptor metabolic process, steroid metabolic process, and amine metabolic process. Of significance, immune-related processes, such as neuroinflammatory response, inflammatory cell apoptotic process, and acute inflammatory response were significantly over-represented.

### Pathway Enrichment Analysis of ADgset and MDDgset

For the ADgset, 153 significantly enriched pathways were identified (Supplementary Table S4), while for MDDgset, 146 significantly enriched pathways were identified (Supplementary Table S5). We obtained 102 shared pathways by integrating pathways enriched in ADgset and MDDgset (Table 2). Beside the shared pathways, there were 95 disease-specific pathways for the two disorders, which included 51 AD-specific pathways and 44 MDD-specific pathways. Among the 102 shared pathways, several pathways were involved in the neural function and neurotransmission, for example, cholinergic synapse, dopaminergic synapse, serotonergic synapse, neuroactive ligand-receptor interaction, and neurotrophin signaling pathway. Some pathways were related to neurological disorders, such as Huntington's disease and AD. At the same time, substance addiction-related pathways were identified, such as amphetamine addiction and cocaine addiction. Metabolism-related pathways such as drug metabolism (cytochrome P450) and tyrosine metabolism were also included, indicating these or related processes may play roles in the pathogenesis of AD and MDD. In addition, about one-third of the shared pathways were related to immune response, immune disease, and pathogens infection, such as inflammatory bowel disease, inflammatory mediator regulation of TRP channels, Th17 cell differentiation, T-cell receptor signaling pathway, Epstein-Barr virus infection, Herpes simplex infection, and HTLV-I infection, suggesting the immune response was involved in the pathogenesis of AD and MDD. Moreover, among the 102 shared pathways, some pathways were related to cell growth and/or survival, including PI3K-Akt signaling, Jak-STAT signaling pathway, MAPK signaling pathway, and mTOR signaling. Among the list, there were also pathways involved in cancer (e.g., bladder cancer, breast cancer, colorectal cancer, endometrial cancer, pancreatic cancer, prostate cancer, and small cell lung cancer), indicating the complicated pathological mechanisms of AD and MDD.

**Table 2:** Pathways commonly enriched in ADgset and MDDgset.

**Module**	**Pathway**	**Degree**	**FDR in ADgset**	**FDR in MDDgset**	**Number of genes**		
					**AD**	**MDD**	**Shared**
Immune response	Hepatitis B	79	8.37 × 10^−05^	1.08 × 10^−05^	19	17	4
	HTLV-I infection	77	2.43 × 10^−03^	7.03 × 10^−06^	23	24	7
	Fluid shear stress and atherosclerosis	76	1.74 × 10^−11^	1.19 × 10^−03^	30	13	5
	Influenza A	76	1.59 × 10^−09^	1.41 × 10^−09^	30	25	8
	Tuberculosis	72	1.43 × 10^−13^	8.03 × 10^−03^	37	13	5
	TNF signaling pathway	69	1.48 × 10^−06^	1.01 × 10^−04^	19	13	5
	Chagas disease (American trypanosomiasis)	68	4.21 × 10^−11^	3.32 × 10^−06^	25	15	7
	Epstein-Barr virus infection	66	4.77 × 10^−05^	3.79 × 10^−03^	24	15	5
	Toll-like receptor signaling pathway	66	5.89 × 10^−05^	6.90 × 10^−05^	16	13	3
	T cell receptor signaling pathway	63	1.20 × 10^−02^	3.10 × 10^−03^	11	10	3
	Hepatitis C	63	6.48 × 10^−04^	1.77 × 10^−04^	16	14	4
	Measles	62	9.96 × 10^−05^	1.46 × 10^−09^	18	22	6
	Toxoplasmosis	60	3.27 × 10^−10^	5.67 × 10^−04^	25	12	3
	Amoebiasis	56	5.45 × 10^−08^	5.39 × 10^−04^	20	11	5
	NOD-like receptor signaling pathway	55	8.97 × 10^−06^	2.40 × 10^−04^	23	16	4
	Inflammatory mediator regulation of TRP channels	52	2.88 × 10^−03^	2.06 × 10^−06^	12	15	3
	Herpes simplex infection	51	2.55 × 10^−08^	6.44 × 10^−05^	29	18	8
	Autophagy - animal	50	5.13 × 10^−04^	3.25 × 10^−02^	16	9	1
	IL-17 signaling pathway	49	7.90 × 10^−07^	1.01 × 10^−04^	18	12	6
	Th17 cell differentiation	49	5.74 × 10^−06^	2.21 × 10^−05^	18	14	4
	Cytokine-cytokine receptor interaction	44	1.04 × 10^−03^	5.53 × 10^−06^	25	25	8
	Leishmaniasis	40	5.49 × 10^−10^	2.49 × 10^−04^	20	10	5
	Inflammatory bowel disease (IBD)	38	3.38 × 10^−09^	1.01 × 10^−04^	18	10	5
	Th1 and Th2 cell differentiation	36	2.03 × 10^−03^	1.48 × 10^−02^	12	8	0
	Rheumatoid arthritis	36	3.38 × 10^−09^	3.14 × 10^−04^	21	11	6
	RIG-I-like receptor signaling pathway	35	2.54 × 10^−03^	1.18 × 10^−02^	10	7	1
	Legionellosis	34	3.34 × 10^−12^	7.58 × 10^−04^	20	8	3
	African trypanosomiasis	32	1.39 × 10^−06^	5.84 × 10^−07^	11	10	4
	Malaria	29	3.27 × 10^−10^	3.62 × 10^−04^	17	8	7
	Graft-versus-host disease	26	7.18 × 10^−06^	1.61 × 10^−02^	11	5	3
	Endocytosis	21	1.11 × 10^−02^	1.38 × 10^−02^	21	16	3
	Antigen processing and presentation	17	4.61 × 10^−04^	5.66 × 10^−03^	12	8	2
	Prion diseases	6	4.35 × 10^−04^	8.85 × 10^−03^	8	5	2
Neurodevelopment	Neurotrophin signaling pathway	67	2.33 × 10^−08^	8.36 × 10^−04^	23	12	6
	Focal adhesion	64	1.36 × 10^−02^	7.74 × 10^−03^	17	14	3
	Glioma	58	4.66 × 10^−03^	3.27 × 10^−06^	9	12	2
	Cholinergic synapse	58	1.18 × 10^−03^	5.62 × 10^−11^	14	22	1
	Dopaminergic synapse	55	2.20 × 10^−04^	1.87 × 10^−18^	17	32	10
	Longevity regulating pathway	53	3.82 × 10^−05^	6.98 × 10^−07^	15	15	3
	Longevity regulating pathway—multiple species	40	1.10 × 10^−03^	4.75 × 10^−09^	10	15	2
	Calcium signaling pathway	37	2.80 × 10^−03^	1.76 × 10^−13^	18	31	3
	Alzheimer's disease	35	2.37 × 10^−17^	7.61 × 10^−04^	41	15	6
	Serotonergic synapse	29	4.49 × 10^−04^	3.36 × 10^−19^	15	31	8
	Amphetamine addiction	24	6.77 × 10^−03^	2.06 × 10^−14^	9	21	5
	Long-term depression	23	3.02 × 10^−02^	5.37 × 10^−05^	7	10	1
	Amyotrophic lateral sclerosis (ALS)	23	9.80 × 10^−06^	2.41 × 10^−06^	12	11	4
	Huntington's disease	14	6.14 × 10^−05^	1.38 × 10^−02^	23	13	5
	Neuroactive ligand-receptor interaction	12	3.00 × 10^−03^	1.33 × 10^−19^	24	47	8
	Cocaine addiction	10	8.31 × 10^−04^	1.44 × 10^−11^	9	16	6
	Drug metabolism—cytochrome P450	4	2.54 × 10^−03^	7.90 × 10^−04^	10	9	3
	Metabolism of xenobiotics by cytochrome P450	3	1.11 × 10^−02^	4.78 × 10^−02^	9	6	1
	Catecholamine biosynthesis, tyrosine = > dopamine = > noradrenaline = > adrenaline	1	3.55 × 10^−02^	7.82 × 10^−04^	2	3	1
Cancer/cell growth	Pathways in cancer	87	1.73 × 10^−09^	1.83 × 10^−07^	48	35	12
	MAPK signaling pathway	81	4.77 × 10^−04^	2.04 × 10^−10^	25	32	9
	Proteoglycans in cancer	79	1.74 × 10^−07^	2.37 × 10^−06^	29	22	6
	PI3K-Akt signaling pathway	75	8.50 × 10^−07^	1.24 × 10^−05^	38	28	7
	HIF-1 signaling pathway	74	2.55 × 10^−08^	1.64 × 10^−08^	21	18	3
	Osteoclast differentiation	70	6.00 × 10^−04^	1.77 × 10^−03^	16	12	4
	Ras signaling pathway	70	5.22 × 10^−04^	7.06 × 10^−04^	23	18	5
	FoxO signaling pathway	68	8.55 × 10^−05^	1.90 × 10^−04^	18	14	5
	Apoptosis	67	1.04 × 10^−06^	2.01 × 10^−02^	22	10	3
	Rap1 signaling pathway	66	2.53 × 10^−03^	3.48 × 10^−06^	20	22	3
	Prostate cancer	63	3.98 × 10^−04^	5.51 × 10^−05^	13	12	2
	cAMP signaling pathway	61	2.58 × 10^−02^	9.35 × 10^−15^	16	34	6
	Pancreatic cancer	61	4.66 × 10^−03^	3.27 × 10^−06^	9	12	5
	EGFR tyrosine kinase inhibitor resistance	60	1.63 × 10^−04^	1.51 × 10^−07^	13	15	3
	mTOR signaling pathway	60	6.16 × 10^−03^	2.00 × 10^−03^	15	13	2
	Jak-STAT signaling pathway	59	1.75 × 10^−02^	2.41 × 10^−06^	14	19	3
	MicroRNAs in cancer	59	1.96 × 10^−03^	2.20 × 10^−02^	26	17	6
	Colorectal cancer	57	2.22 × 10^−04^	5.43 × 10^−03^	11	7	3
	Choline metabolism in cancer	54	9.21 × 10^−03^	1.77 × 10^−04^	11	12	2
	Small cell lung cancer	50	8.61 × 10^−03^	2.77 × 10^−02^	10	7	2
	Breast cancer	49	2.11 × 10^−02^	1.35 × 10^−04^	13	15	4
	Non-small cell lung cancer	45	2.59 × 10^−02^	2.15 × 10^−04^	7	9	2
	cGMP-PKG signaling pathway	43	5.19 × 10^−03^	1.35 × 10^−05^	16	18	1
	Endometrial cancer	40	3.76 × 10^−03^	2.00 × 10^−03^	8	7	2
	AMPK signaling pathway	36	5.00 × 10^−07^	8.96 × 10^−03^	21	10	2
	p53 signaling pathway	23	2.42 × 10^−03^	1.11 × 10^−02^	10	7	2
	Bladder cancer	23	1.15 × 10^−03^	6.87 × 10^−04^	8	7	3
	Chemical carcinogenesis	3	2.53 × 10^−03^	8.05 × 10^−03^	11	8	1
Endocrine	AGE-RAGE signaling pathway in diabetic complications	76	4.26 × 10^−07^	1.06 × 10^−05^	19	14	6
	Non-alcoholic fatty liver disease (NAFLD)	71	2.82 × 10^−12^	1.32 × 10^−02^	32	11	6
	Sphingolipid signaling pathway	67	2.28 × 10^−04^	7.74 × 10^−03^	16	10	3
	Endocrine resistance	65	8.55 × 10^−05^	7.19 × 10^−09^	15	18	5
	Insulin resistance	65	3.23 × 10^−07^	4.06 × 10^−03^	20	10	3
	Prolactin signaling pathway	61	2.54 × 10^−03^	3.70 × 10^−05^	10	11	3
	Phospholipase D signaling pathway	59	2.78 × 10^−04^	3.48 × 10^−06^	18	18	1
	Acute myeloid leukemia	52	2.04 × 10^−02^	7.58 × 10^−04^	7	8	1
	Chronic myeloid leukemia	49	8.82 × 10^−03^	8.62 × 10^−04^	9	9	4
	VEGF signaling pathway	46	2.80 × 10^−03^	5.03 × 10^−03^	9	7	2
	GnRH signaling pathway	45	3.64 × 10^−02^	1.07 × 10^−06^	9	15	1
	Apelin signaling pathway	45	6.89 × 10^−03^	6.51 × 10^−06^	14	17	1
	Adipocytokine signaling pathway	43	9.10 × 10^−06^	3.01 × 10^−03^	14	8	3
	Type II diabetes mellitus	34	2.42 × 10^−03^	6.12 × 10^−03^	8	6	1
	Type I diabetes mellitus	27	2.60 × 10^−08^	1.51 × 10^−04^	14	8	2
	Regulation of lipolysis in adipocytes	27	4.09 × 10^−04^	1.30 × 10^−04^	10	9	3
	Ovarian steroidogenesis	22	1.94 × 10^−07^	2.00 × 10^−03^	14	7	1
	Aldosterone-regulated sodium reabsorption	20	4.12 × 10^−02^	4.78 × 10^−02^	5	4	0
	Antifolate resistance	20	1.96 × 10^−04^	5.43 × 10^−03^	8	5	4
	Renin secretion	16	1.56 × 10^−02^	1.91 × 10^−05^	8	11	2
	Tyrosine metabolism	4	9.19 × 10^−03^	1.65 × 10^−03^	6	6	4
Not included	GABA biosynthesis, eukaryotes, putrescine = > GABA	NA	1.33 × 10^−02^	5.02 × 10^−03^	3	3	2

### Construction of the Interaction Network of Pathway-Pathway-Genes

**Figure 2:**
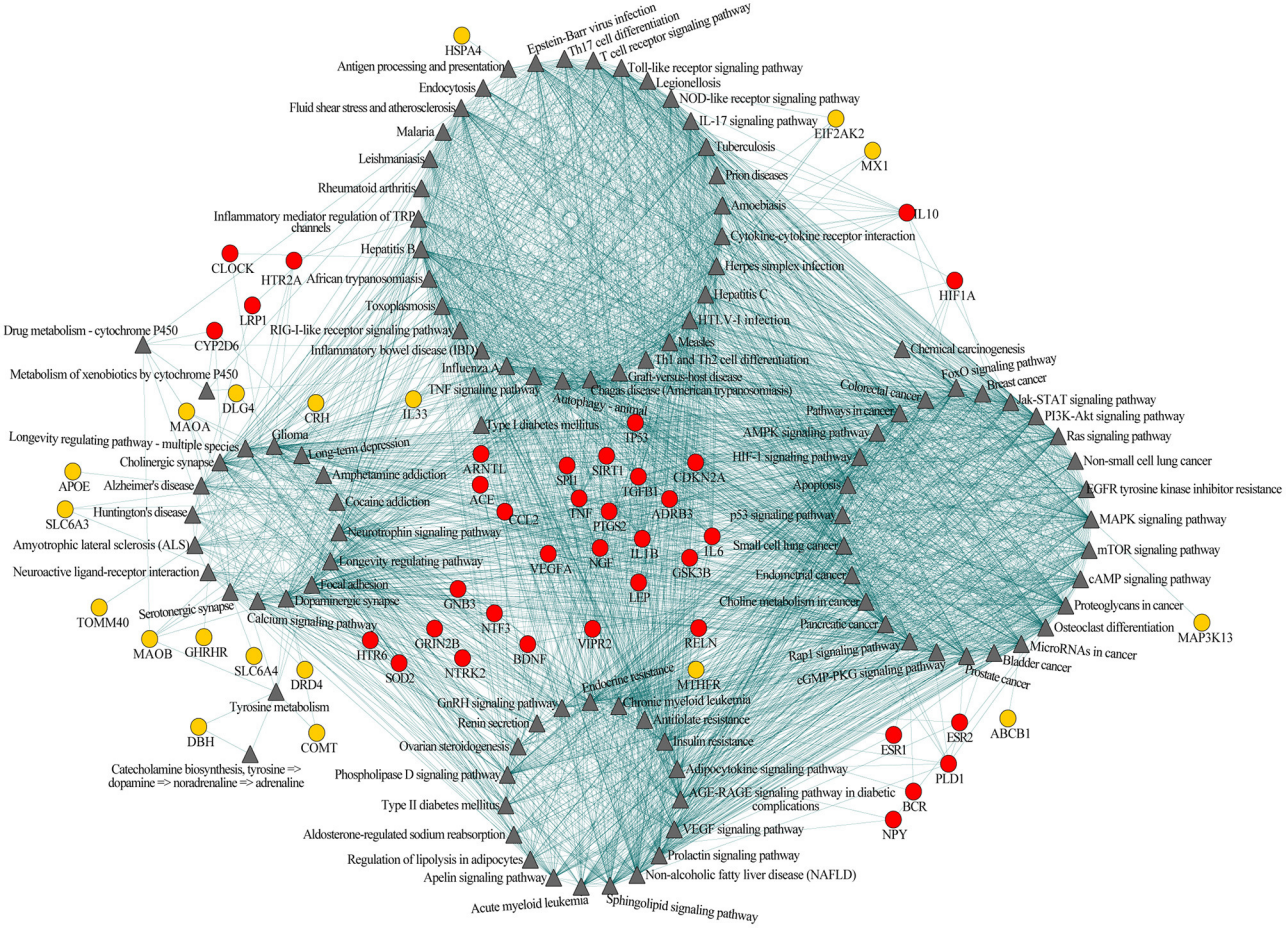
Pathway-pathway-shared genes network of 101 shared pathways and 56 shared genes of Alzheimer's disease (AD) and major depressive disorder (MDD). Gray triangular nodes represent pathways, red circular nodes represent genes located within two or more modules, and orange circular nodes represent genes located within one module. Edges represent connections between pathway-pathway and pathway-shared genes.

Among the 102 shared pathways, 101 pathways, except the gamma-aminobutyric acid (GABA) biosynthesis, are included in the cross-talk analysis. Furthermore, we added the shared genes into the pathway-pathway interaction network to form the interaction network of pathway-pathway-shared genes to explore the biological function in which shared genes participated. Of the 77 shared genes, 56 were included in 99 shared pathways (except aldosterone-regulated sodium reabsorption, GABA biosynthesis and Th1 and Th2 cell differentiation). This network consisted of 2,767 edges (connections) and 157 nodes (pathways and shared genes) (Figure 2). Of the edges, 2,380 were connections between pathways, and the other 387 were connections between pathways and the shared genes. Among the 157 nodes, there were 101 pathways (Figure 2, triangular node) and 56 shared genes (Figure 2, circular nodes).

Based on the biological function, this network could be largely divided into four modules, i.e., immune, neural, cancer, and endocrine (Figure 2). The first module was mainly related to immunological regulation, immunological disorders, and pathogens infection (e.g., antigen processing and presentation, T-cell receptor signaling pathway and Th17 cell differentiation, tumor necrotic factor (TNF) signaling pathway, inflammatory bowel disease, influenza A, rheumatoid arthritis, Epstein-Barr virus infection, and herpes simplex infection). The second module primarily included pathways related to neuronal function, substance addiction, and neurological disorders (e.g., cholinergic synapse, dopaminergic synapse, serotonergic synapse, amphetamine addiction, cocaine addiction, Alzheimer's disease, and Huntington's disease). The major theme of the third module was cell growth and tumorigenesis (e.g., MAPK signaling pathway, PI3K-Akt signaling pathway, bladder cancer, breast cancer, pancreatic cancer, and prostate cancer). The last module was largely concentrated in the regulation of the endocrine system, like endocrine resistance and renin secretion. Obviously, these four modules were not independent of each other; instead, they were connected through one or more pathways, indicating the existence of an immune-neuronal-cell development-endocrine regulatory network underlying the mechanisms of AD and MDD. Meanwhile, some of the 56 shared genes mainly participated in the regulation within one module (Figure 2, orange circular nodes), but others participated in the regulation of two or more modules (Figure 2, red circular nodes). The shared genes between two or more modules may play essential roles in maintaining the balance of different systems.

**Figure 3:**
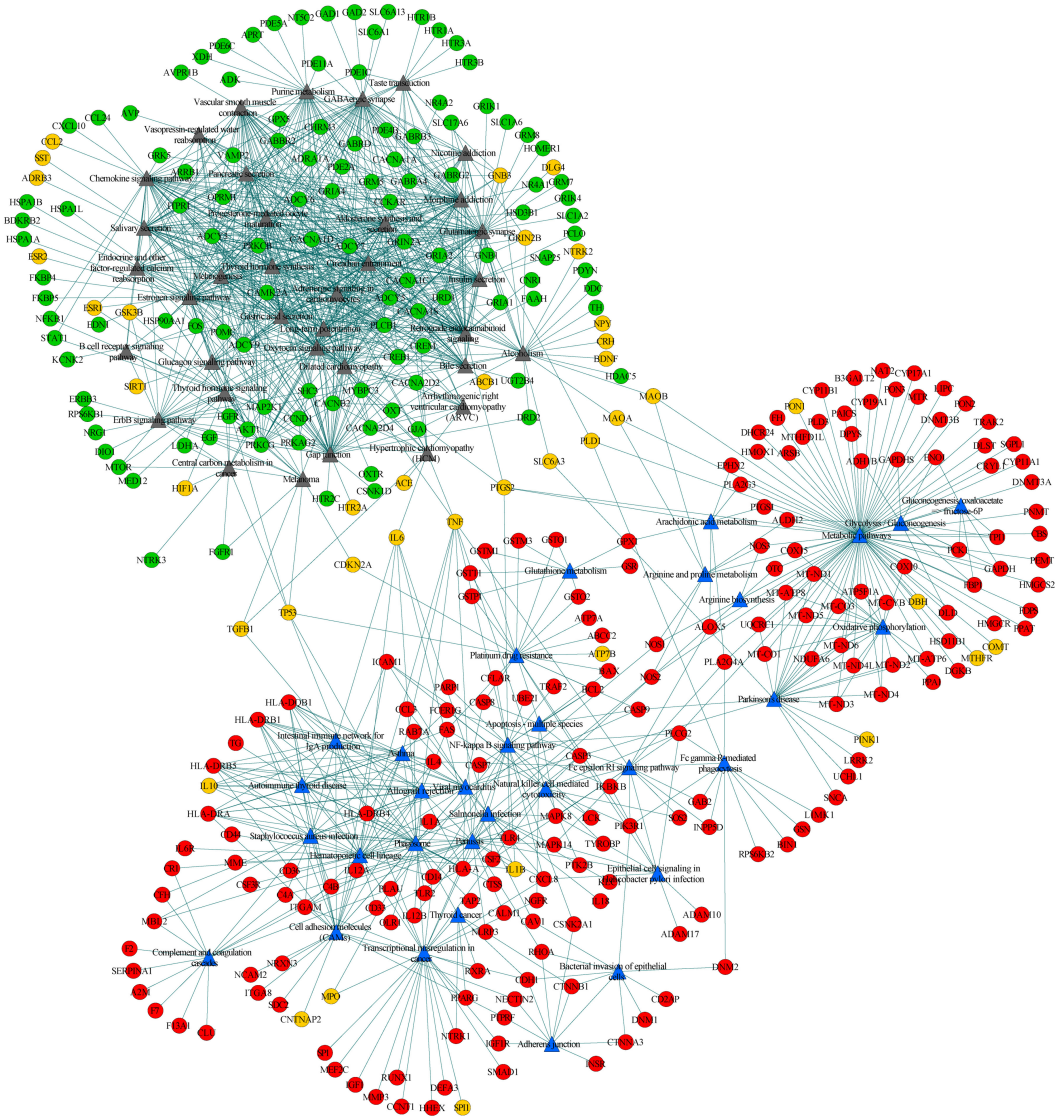
Pathway-pathway-genes network of disease-specific pathways and their linked genes. Blue triangular nodes represent AD-specific pathways, gray triangular nodes represent MDD-specific pathways, red circular nodes represent AD-specific genes, green circular nodes represent MDD-specific genes, and orange circular node represents shared genes. Edges represent connections between pathway-pathway and pathway-genes.

To further explore the connections between disease-specific pathways and their linked genes, we constructed a network for AD-specific pathways, MDD-specific pathways, and their linked genes. Among the 95 disease-specific pathways (51 AD-specific and 44 MDD-specific pathways), 68 (32 AD-specific and 36 MDD-specific pathways) met the criterion of pathway cross talk. The constructed network contained 1,371 edges and 410 nodes (pathways and genes) (Figure 3). Of the edges, 386 were connections between pathways and 985 were connections between pathways and genes. The 410 nodes included 32 AD-specific pathways (Figure 3, blue triangular node), 36 MDD-specific pathways (Figure 3, gray triangular node), 186 AD-associated genes (Figure 3, red circular nodes), 117 MDD-associated genes (Figure 3, green circular nodes), and 39 shared genes (Figure 3, orange circular nodes). This network could be divided into two modules, namely, the upper left module that included the MDD pathways and their connected genes, and the lower right module that included the AD pathways and their connected genes. The two modules were linked by several shared genes.

### Analysis of New Candidate Genes Related to AD and MDD

**Figure 4:**
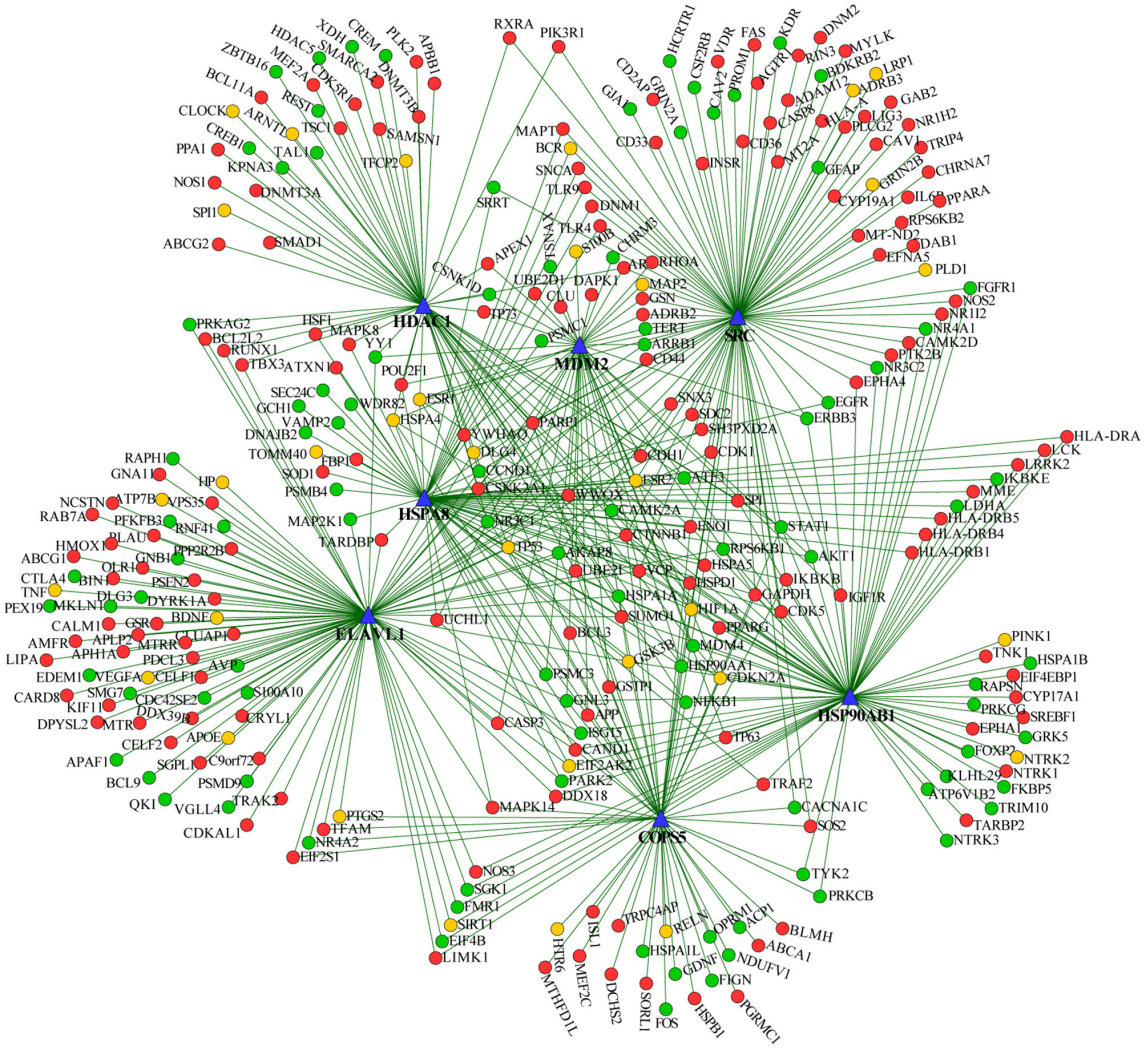
Seven predicted novel candidate genes and known genes connected with them. Triangular nodes are predicted novel candidate genes, red nodes are AD-associated genes, green nodes are MDD-associated genes, and orange nodes are shared genes.

We extracted the overlapping part of the AD-specific network and the MDD-specific network, which contained 3,001 nodes and 4,900 interactions. Among the nodes, 192 were genes from ADgset, 106 were genes from MDDgset, and 74 were shared genes. There were 2,629 first neighbor nodes of the 77 shared genes, from which we identified the novel candidate genes *via* the “guilt-by-association” principle (Oliver, 2000). That is, a node tends to participate in the same or similar cellular functions if the majority of its neighbors in the interactome network associated with specific cellular functions (e.g., a certain disease or phenotype). Ranking in descending order of node degree, we found 37 novel candidate genes (Table 3) directly interacted with 6 or more of the 77 shared genes. Furthermore, we selected 7 novel candidate genes directly interacted with 9 or more of the 77 shared genes, including ELAV like RNA-binding protein 1 (*ELAVL1*), heat shock protein 90 alpha family class B member 1 (*HSP90AB1*), heat shock protein family A (*HSPA8*), MDM2 proto-oncogene (*MDM2*), SRC proto-oncogene, non-receptor tyrosine kinase (*SRC*), COP9 signalosome subunit 5 (*COPS5*), and HDAC1 histone deacetylase 1 (*HDAC1*). By mapping these seven genes with the most neighbors to the disease network, we could retrieve their specific interaction network (Figure 4), which consisted of 306 nodes (genes) and 522 edges (connections). Besides the seven novel candidate genes (Figure 4, triangular nodes), this network also included 168 AD-associated genes (Figure 4, red circular nodes) and 98 MDD-associated genes (Figure 4, green circular nodes) and 33 shared genes (Figure 4, orange circular nodes). These seven novel candidate genes were closely related to AD-associated genes, MDD-associated genes, and shared genes, indicating the credibility of our results. Above all, they were expected to provide guidance for clinical and basic medical researches.

**Table 3:** Novel candidate genes potentially related to AD and MDD.

**Gene ID**	**Gene symbol**	**Node degree**	**Gene name**
1994	ELAVL1	12	ELAV like RNA binding protein 1
3326	HSP90AB1	10	Heat shock protein 90 alpha family class B member 1
3312	HSPA8	10	Heat shock protein family A
4193	MDM2	10	MDM2 proto-oncogene
6714	SRC	10	SRC proto-oncogene, non-receptor tyrosine kinase
10987	COPS5	9	COP9 signalosome subunit 5
3065	HDAC1	9	Histone deacetylase 1
3725	JUN	8	Jun proto-oncogene, AP-1 transcription factor subunit
60	ACTB	8	Actin beta
2033	EP300	8	E1A binding protein p300
2534	FYN	8	FYN proto-oncogene, Src family tyrosine kinase
3066	HDAC2	8	Histone deacetylase 2
5578	PRKCA	8	Protein kinase C alpha
5970	RELA	8	RELA proto-oncogene, NF-kB subunit
4088	SMAD3	8	SMAD family member 3
4904	YBX1	8	Y-box binding protein 1
1051	CEBPB	7	CCAAT enhancer binding protein beta
8841	HDAC3	7	Histone deacetylase 3
4609	MYC	7	MYC proto-oncogene, bHLH transcription factor
6774	STAT3	7	Signal transducer and activator of transcription 3
203068	TUBB	7	Tubulin beta class I
1869	E2F1	6	E2F transcription factor 1
2885	GRB2	6	Growth factor receptor bound protein 2
10524	KAT5	6	Lysine acetyltransferase 5
4067	LYN	6	LYN proto-oncogene, Src family tyrosine kinase
4130	MAP1A	6	Microtubule associated protein 1A
5594	MAPK1	6	Mitogen-activated protein kinase 1
4869	NPM1	6	Nucleophosmin 1
5499	PPP1CA	6	Protein phosphatase 1 catalytic subunit alpha
5501	PPP1CC	6	Protein phosphatase 1 catalytic subunit gamma
5566	PRKACA	6	Protein kinase cAMP-activated catalytic subunit alpha
5925	RB1	6	RB transcriptional corepressor 1
6464	SHC1	6	SHC adaptor protein 1
4087	SMAD2	6	SMAD family member 2
10273	STUB1	6	STIP1 homology and U-box containing protein 1
6613	SUMO2	6	Small ubiquitin like modifier 2
7534	YWHAZ	6	Tyrosine 3-monooxygenase/tryptophan 5-monooxygenase activation protein zeta

**Figure 5:**
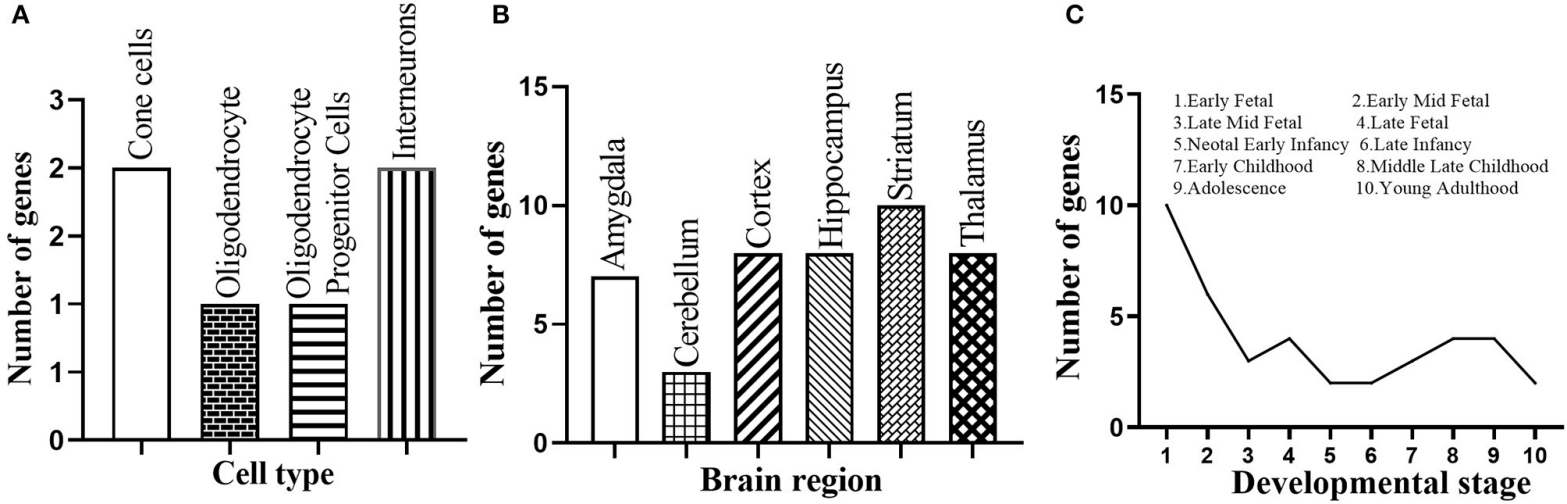
Spatio-temporal expression of the 37 novel candidate genes. **(A)** Number of the novel candidate genes with substantial overrepresentation in brain cell types. **(B)** Number of the novel candidate genes with substantial overrepresentation in brain regions. **(C)** Number of the novel candidate genes expressed in specific developmental period.

We further performed a spatio-temporal and cell type-specific expression analysis on the 37 new candidate genes. Of which, 28 genes could be detected in public available brain cell type expression dataset CSEA, and 6 of them (i.e., *E2F1, RELA, MAPK1, FYN, CEBPB, STAT3*) showed substantial overrepresentation (pSI = 0.05, Supplementary Table S6) in cone cells, oligodendrocytes, interneurons, and others (Figure 5A). In addition, all the 37 genes expressed in the brain regions of different development stages, and 20 genes (i.e., *ACTB, CEBPB, COPS5, E2F1, ELAVL1, HDAC2, JUN, LYN, MAP1A, MAPK1, MYC, NPM1, PPP1CC, PRKCA, RB1, SMAD3, SRC, SUMO2, TUBB, YBX1*) showed substantial overrepresentation under the threshold pSI = 0.05 (Supplementary Table S7). Striatum, hippocampus, cortex, and thalamus contained 10, 8, 8, and 8 novel candidate genes under pSI = 0.05, respectively (Figure 5B). Further, the number of genes was generally fewer after birth (Figure 5C). In addition, we searched the expression patterns of 37 novel candidate genes in Human Brain Transcriptome (HBT) database (www.humanbraintranscriptome.org). We found that the expression of most genes changed with different developmental stages, which are specifically manifested in a downward trend after birth ((Supplementary Figures 1A–D) and an upward trend after birth (Supplementary Figures 1E–H). Thus, most of these novel candidate genes were significantly expressed in the brain or brain cell lines and changed with different ages, indicating they could play important roles in the normal function of the brain.

## Discussion

In the past decades, our capability in identifying genes related to complex disease like AD and MDD has been greatly improved. However, complex diseases are rarely a straight-forward dysfunction in a specific gene, but rather a consequence of the collective interplay of multiple genes (Menche et al., 2015). While previous studies have reported the neurobiology and susceptibility gene correlations between MDD and neurodegenerative diseases like AD, the underlying mechanisms still need to be explored. The analysis on the susceptibility gene of AD and MDD from the aspect of systems biology could provide insight into the comorbidity of these two diseases beyond studies based on single risk gene. In current research, we analyzed the functions of genes associated with AD and MDD and deciphered the interrelationship between these genes. In addition, we predicted the novel candidate genes potentially related to AD and MDD.

Based on 650 AD-associated genes and 447 MDD-associated genes, we retrieved 77 shared genes between AD and MDD. Then, GO biological processes enriched in the 77 shared genes and pathways enriched in AD-associated genes or MDD-associated genes were detected. Biological processes such as response to substances (e.g., toxic substances, hormones, oxidative stress, and alcohol), cognition, regulation of neurogenesis, aging, and regulation of developmental growth were enriched in the shared genes, indicating the significance of these processes in AD and MDD pathology. Among the 102 overlapping pathways, AD was the most significant one, indicating some AD susceptibility genes are MDD genes. Other pathways were related to neuroactive ligand-receptor interaction, synapse (e.g., serotonergic, dopaminergic, and cholinergic), and substance addictions (e.g., amphetamine and cocaine). Neuroactive ligand-receptor interaction, as a neurodevelopment-related pathway, has been implicated in AD and MDD. Synaptic dysfunction can lead to two important symptoms of AD and MDD, i.e., cognitive impairment and memory loss, indicating the importance of synapse-related pathways in pathophysiology of these two disorders (Boyle et al., 2010; Sierksma et al., 2010). Consistently, several shared pathways were related to immune responses (e.g., Th1 and Th2 cell differentiation and Th17 cell differentiation) and immune diseases (e.g., inflammatory bowel disease and type I diabetes). For example, Th17 participated in neuroinflammation and neurodegeneration of AD by regulating inflammatory cytokines and signaling (Zhang et al., 2013). In addition, a predominant Th1/Th17 inflammatory response in placental immune cells plays a crucial role in regulating depressive symptoms during pregnancy (Leff-Gelman et al., 2016). It has been showed the involvement of neuroinflammation in AD and MDD pathological mechanisms, in which the complement molecules exert indispensable efforts (Lian et al., 2016; Yao and Li, 2020). Studies have shown that the microbial imbalance of the intestinal flora may explain the association between inflammatory bowel disease and cognitive function of AD or MDD (Bonaz and Bernstein, 2013; Chen et al., 2016; Gareau, 2016; Spielman et al., 2018). Furthermore, it has been indicated that diabetes can cause deterioration of cognitive function and increase the risk of dementia and depression (Szatmári et al., 2017). Moreover, infection-related pathways were also enriched in our disease-related genes, such as Epstein-Barr virus (EBV) infection and herpes simplex virus (HSV) infection. Previous studies have reported HSV-1 infection may impair the hippocampal function and is a negative factor of AD (Li Puma et al., 2019). EBV has also been reported to be related to the cognitive decline in patients with AD (Shim et al., 2017). In addition, patients with MDD have altered levels and patterns of EBV antibodies indicating a therapeutic intervention targeting EBV available for MDD individuals (Jones-Brando et al., 2020). In our study, virus infection like HSV and EBV infection included in the shared pathway list, indicating that virus infection may increase the risk of AD and MDD. In the future, we should explore the impact of viral infections on the clinical treatment of AD and MDD. Moreover, some pathways involved in cancers (e.g., prostate cancer, glioma, and pancreatic cancer) and their developmental processes (e.g., apoptosis) were also existed in 102 shared pathways. Researchers have revealed that multitudinous cancer survivors had lower AD and MDD risk (Caruso et al., 2017; Frain et al., 2017). Our shared pathways include some cancer and related pathways, suggesting that cancer and/or cancer treatment may affect AD and MDD progression. In the pathway cross-talk analysis, the pathway-pathway-shared genes interaction network containing 2,767 edges (connections) and 157 nodes (pathways and shared genes). Pathways are tightly connected in this network and may collectively influence the occurrence of AD and MDD. In sharp contrast with the network between shared pathways, in the network of disease-specific pathways, the pathways between AD and MDD were only sparsely connected through a few shared genes.

We further identified 37 new genes highly interconnected with the 77 shared genes, which were also potentially related to AD and MDD. Of which, the node degrees of *ELAVL1, HSP90AB1, HSPA8, MDM2, SRC, COPS5*, and *HDAC1* were the highest with degrees >8.0. ELAVL1 (also known as HuR/HuA), an RNA-binding protein, belongs to the ELAV/Hu family and is ubiquitously expressed in all human tissues (Fan and Steitz, 1998). ELAVL1 plays a critical role in stress-induced synaptic dysfunction (He et al., 2019) and contributes to neuroinflammation in *ELAVL1*-knockout mouse model (Chen et al., 2017). It has been showed that neuroinflammation and stress are involved in AD and MDD pathological mechanisms (Bolos et al., 2017; Ignácio et al., 2019; Sotiropoulos et al., 2019). *ELAVL1* may promote the progress of AD and MDD through regulating neuroinflammatory and stress-mediated synaptic function. Whether it can be used as a potential therapeutic target for AD, MDD, and their comorbidity in the future remains to be further explored. HSP90AB1 and HSPA8 are heat shock proteins (HSPs), and disruption of HSPs expression has been described as a possible mechanism in the etiology and progression of AD (Gorenberg and Chandra, 2017). HSPA8 is a member of HSP70 family proteins that may affect the action of antidepressants and thus their therapeutic efficacy in treatment of MDD (Pae et al., 2007). Our results suggest that HSPs are expected to be potential intervention targets for the treatment of the comorbidity underlying AD and MDD. *MDM2* gene is a proto-oncogene, and its expression mediates important cancer pathology (Oliner et al., 2016). Our pathway cross-talk analysis showed the close interlink between the neural and cancer models. The involvement of *MDM2* in comorbidity of AD and MDD may be related to tumorigenesis. COPS5 is an evolutionarily conserved and multifunctional protein and its overexpression in mice brain significantly increased amyloid-β protein levels in the cortex and hippocampus (Wang et al., 2015). As we all know, amyloid-β takes part in the pathogenesis of both AD and MDD (Blennow et al., 2006; Pomara et al., 2012). The activation of HDAC1, a histone deacetylase 1, has shown therapeutic potential against functional decline in brain aging and neurodegeneration (Pao et al., 2020). It has been reported that HDAC1 is involved in fluoxetine-mediated antidepressant mechanism in the LPS-induced depression mouse model (Li et al., 2021). Although there are few reports that indicate its role of HDAC1 in the comorbidity processes of AD and MDD, histone modifications are widely involved in the gene expression regulation. Our PPI results show that HDAC1 is closely interlinked with shared genes of AD and MDD, implying that HDAC1 has potential regulatory functions on the shared genes. To verify the results, we further checked the expression pattern of these 37 novel candidate genes in brain tissues and cell lines. As expected, most of the novel candidate genes were significantly expressed in the brain.

There were several limitations in this study. For instance, our disease susceptibility genes were mainly collected from currently available studies and public databases, which should be deemed as incomplete as our exploration on the molecular features of AD and MDD is still ongoing. In future, more genes involved in these disorders may be identified, and some false-positive genes may be excluded. Similarly, the protein-protein interaction databases and the human interactome constructed in this work should be treated in the same way.

## Conclusion

Based on the available susceptibility genes related to AD or MDD, we analyzed the biochemical pathways and the interactions between genes and pathways potentially involved in the comorbidity of the two disorders. We found that biological processes related to metabolism, immune response, cancer, and neural disease were enriched in 77 shared genes. And there existed four interconnected modules between 102 shared pathways, namely, immune, neuro, cancer, and endocrine. Of significance, we identified 37 interesting novel candidate genes related to AD and MDD based on the PPI analysis. Such analysis of AD- and MDD-related genes will not only improve our understanding of the mechanisms or their comorbidity but also help us to explore new biomarkers for the comorbidity of these diseases.

## Data Availability Statement

The original contributions presented in the study are included in the article/Supplementary Material, further inquiries can be directed to the corresponding authors.

## Author Contributions

PG and SC designed the experiments. PG, HW, and JW performed the experiments and data analysis. PG, YW, and JW wrote the manuscript. All authors have read and approved the final manuscript.

## Funding

This study was supported in part by grants from National Key Research and Development Program of China (No. 2016YFC0906300) and National Natural Science Foundation of China (Nos. 91746205 and 31271411).

## Conflict of Interest

The authors declare that the research was conducted in the absence of any commercial or financial relationships that could be construed as a potential conflict of interest.

## Publisher's Note

All claims expressed in this article are solely those of the authors and do not necessarily represent those of their affiliated organizations, or those of the publisher, the editors and the reviewers. Any product that may be evaluated in this article, or claim that may be made by its manufacturer, is not guaranteed or endorsed by the publisher.

## Supplementary Material

Supplementary Figure 1 The expression pattern of some novel candidate genes in specific developmental period. Some genes are specifically manifested in a downward trend after birth **(A–D)** and an upward trend after birth **(E–H)**. The y-axis represents the intensity of gene expression, and x-axis represents age. Periods 1–7 represent postconceptional age and 8–15 represent postnatal years. NCX, neocortex; HIP, hippocampus; AMY, amygdala; ST, striatum; MD, mediodorsal nucleus of the thalamus; CBC, cerebellar cortex.

Supplementary Material 1 The background PPIN constructed in the current study.

Supplementary Table S1 List of 650 genes associated with Alzheimer's Disease.

Supplementary Table S2 List of 447 genes associated with major depressive disorder.

Supplementary Table S3 The GO biological processes significantly enriched in the 77 shared genes between AD and MDD.

Supplementary Table S4 153 pathways significantly enriched in the 650 AD-associated genes.

Supplementary Table S5 146 pathways significantly enriched in the 447 MDD-associated genes.

Supplementary Table S6 Expression of novel candidate genes in brain cells.

Supplementary Table S7 Expression of novel candidate genes in brain region and developmental stages.
