# Supplementary figures and images for: A Systematic Analysis on the Genes and Their Interaction Underlying the Comorbidity of Alzheimer's Disease and Major Depressive Disorder
Source: Front Aging Neurosci. 2022 Jan 20;13:789698. doi: 10.3389/fnagi.2021.789698 (PMC8810513; doi:10.3389/fnagi.2021.789698)

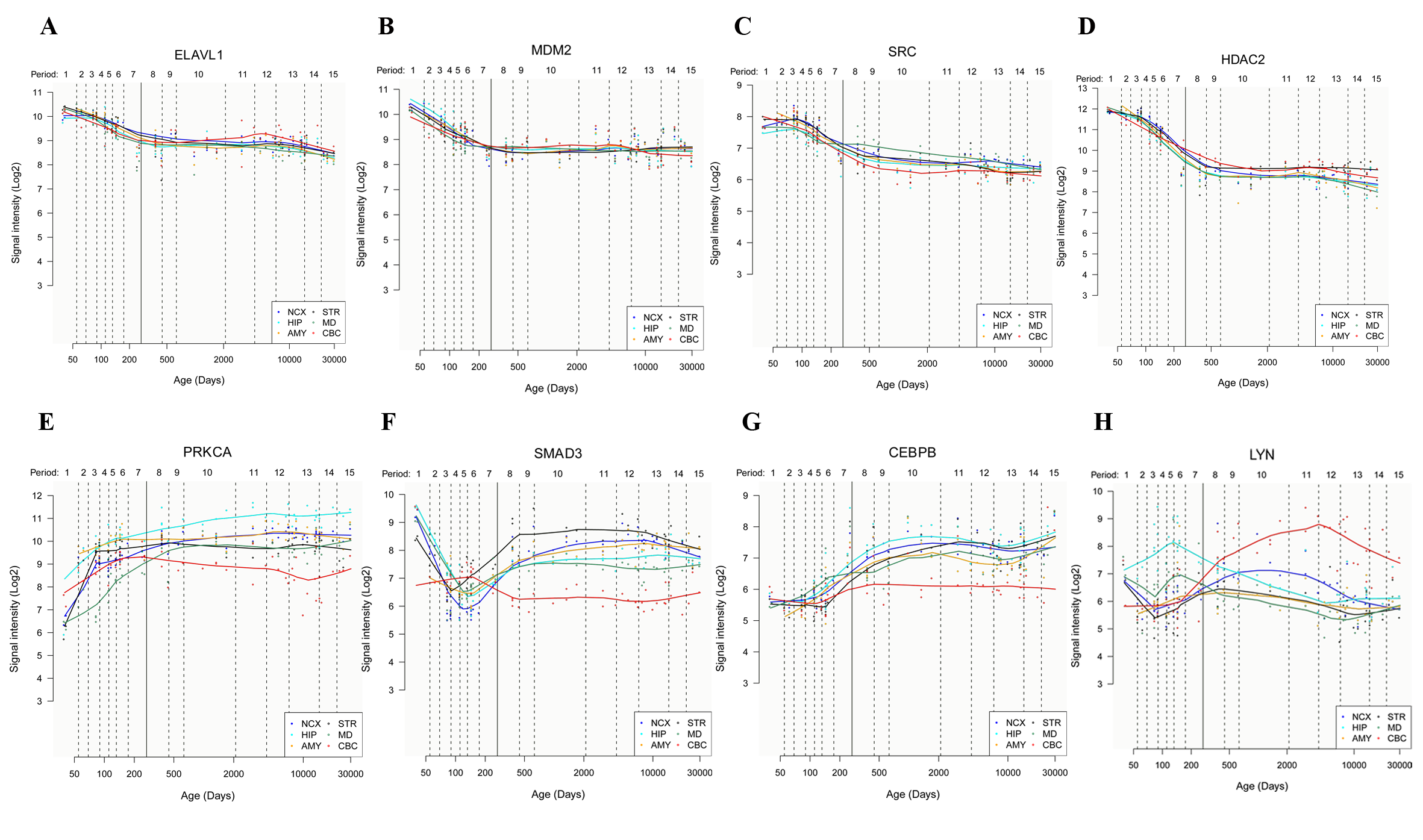

Supplement: Supplementary Figure 1 — The expression pattern of some novel candidate genes in specific developmental period. Some genes are specifically manifested in a downward trend after birth (A–D) and an upward trend after birth (E–H). The y-axis represents the intensity of gene expression, and x-axis represents age. Periods 1–7 represent postconceptional age and 8–15 represent postnatal years. NCX, neocortex; HIP, hippocampus; AMY, amygdala; ST, striatum; MD, mediodorsal nucleus of the thalamus; CBC, cerebellar cortex. [file Image_1.tif]
